# First-principles study of Li-doped planar g-C_3_N_5_ as reversible H_2_ storage material

**DOI:** 10.3389/fchem.2023.1301690

**Published:** 2023-10-25

**Authors:** Xihao Chen, Zonghang Liu, Jiang Cheng, Jiwen Li, Donglin Guo, Liang Zhang, Xianghong Niu, Ning Wang, Guangzhao Wang, Peng Gao

**Affiliations:** ^1^ State Key Laboratory of Precision Spectroscopy, East China Normal University, Shanghai, China; ^2^ School of Materials Science and Engineering, Chongqing University of Arts and Sciences, Chongqing, China; ^3^ Chongqing Key Laboratory of Precision Optics, Chongqing Institute of East China Normal University, Chongqing, China; ^4^ School of Science and Engineering, Shenzhen Key Laboratory of Functional Aggregate Materials, The Chinese University of Hong Kong, Shenzhen, China; ^5^ College of Physics and Electronic Engineering, Northwest Normal University, Lanzhou, China; ^6^ School of Electric and Electrical Engineering, Shangqiu Normal University, Shangqiu, China; ^7^ New Energy Technology Engineering Laboratory of Jiangsu Province, School of Science, Nanjing University of Posts and Telecommunications, Nanjing, China; ^8^ Key Laboratory of Extraordinary Bond Engineering and Advanced Materials Technology of Chongqing, School of Electronic Information Engineering, Yangtze Normal University, Chongqing, China; ^9^ School of Chemistry and Molecular Bioscience, University of Wollongong, Wollongong, NSW, Australia; ^10^ Molecular Horizons, University of Wollongong, Wollongong, NSW, Australia

**Keywords:** hydrogen storage, reversible, g-C_3_N_5_, Li-decorated, DFT

## Abstract

Under the background of energy crisis, hydrogen owns the advantage of high combustion and shows considerable environment friendliness; however, to fully utilize this novel resource, the major hurdle lies in its delivery and storage. The development of the in-depth yet systematical methodology for two-dimensional (2D) storage media evaluation still remains to be challenging for computational scientists. In this study, we tried our proposed evaluation protocol on a 2D material, g-C_3_N_5_, and its hydrogen storage performance was characterized; and with addition of Li atoms, the changes of its electronical and structural properties were detected. First-principles simulations were conducted to verify its thermodynamics stability; and, its hydrogen adsorption capacity was investigated qualitatively. We found that the charges of the added Li atoms were transferred to the adjacent nitrogen atoms from g-C_3_N_5_, with the formation of chemical interactions. Thus, the isolated metallic sites tend to show considerable electropositivity, and can easily polarize the adsorbed hydrogen molecules, and the electrostatic interactions can be enhanced correspondingly. The maximum storage capacity of each primitive cell can be as high as 20 hydrogen molecules with a gravimetric capacity of 8.65 wt%, which surpasses the 5.5 wt% target set by the U.S. Department of Energy. The average adsorption energy is ranged from −0.22 to −0.13 eV. We conclude that the complex 2D material, Li-decorated g-C_3_N_5_ (Li@C_3_N_5_), can serve as a promising media for hydrogen storage. This methodology provided in this study is fundamental yet instructive for future 2D hydrogen storage materials development.

## 1 Introduction

Under the context of global energy crisis, development of clean yet renewable resource is highly needed. Hydrogen, which is regarded as the fuel of future, owns the advantage of high renew-ability, and its combustion has zero-emission of CO_2_ (Allendorf et al., 2018; Züttel, 2004; U.S Department of Energy, 2020); thus it can be widely applied in many fields. However, in real practice, the main limitation lies in the fact that we are lack of high-efficiency storage media (U.S Department of Energy, 2020; Huang and Autrey, 2012; Züttel, 2003). Solid state materials based media are preferred due to their superior properties. In the current stage, some researchers tend to use metal-organic frameworks (MOFs) or metal hydrides based materials for this task (Bogdanovi and Schwickardi, 1997; Züttel et al., 2003; Sakintuna et al., 2007; Graetz, 2009; Gao et al., 2022); and at the same time, hydrocarbons or BN compounds had also been reported to own high content of hydrogen (Campbell et al., 2010; Luo et al., 2011a; Luo et al., 2011b; Teichmann et al., 2012; Gao et al., 2020; Gao and Zhang, 2020; Gao and Zhang, 2021). To successfully develop a high-performance hydrogen storage media, not only the storage capacity should be highlighted, manufacturing convenience is also crucial.

Within the past few decades, 2D carbon materials based storage media are promisingly emerging due to their high adsorption ability (Jürgens et al., 2003; Holst and Gillan, 2008; Thomas et al., 2008; Wei and Jacob, 2013; Algara-Siller et al., 2014; Dong et al., 2014; Fina et al., 2015; Hussain et al., 2016; Liu et al., 2019; Wang et al., 2019; Gao et al., 2021a; Wang et al., 2023; Zhang et al., 2023). These kinds of materials own superior aperture structures that enable themselves to be easily doped metal atoms or superalkali clusters for properties optimization and performance enhancement (Zhang et al., 2009; Wu et al., 2013; Zhu et al., 2014; Mahmood et al., 2015; Nair et al., 2015; Ruan et al., 2015; Wei et al., 2016; Gao et al., 2019; Panigrahi et al., 2020; Gao et al., 2021b; Gao et al., 2021c; Chen et al., 2021). Moreover, their original structure features can be qualitatively correlated with their optimizability and functions; thus computational investigation and evaluation of the novel derivatization units can be highly instructive for experimental practice (Wang et al., 2018; Bafekry et al., 2021). The graphene-like 2D unit, g-C_3_N_5_, is reported to be one of the most promising structures for electronic and optical devices development; and, it is proposed to own higher adsorption carrier, making itself suitable for storage media design.

Moreover, a systematic computation protocol for 2D materials’ hydrogen storage performance evaluation is of great importance for future studies; in this study, we proposed a density functional theory (DFT) calculations based methodology for the solution of structural and electronic properties of the pristine and metal-doped 2D materials, and the rules of the correlation between the properties and functions were summarized as reference. We tried our methodology upon Li@C_3_N_5_; and its hydrogen adsorption mechanism was successfully summarized. We anticipate that the fundamental insights provided in this study will be highly instructive for future development of 2D energy storage media.

## 2 Computational details

First-principles calculations were carried out within the Vienna Ab-initio Simulation Package (VASP) under periodic boundary conditions (Kresse and Furthmüller, 1996; Grimme, 2006). Considering the balance between cost and efficiency, g-C_3_N_5_ primitive cell was selected as a prototype, and the applied lattice parameters were *a* = *b* = 15 Å. The electronic states were explored with the plane wave basis projector augmented wave (PAW) method (Blöchl, 1994), the cutoff of energy is set to 520 eV. The exchange-correlation energies were obtained with the generalized gradient approximation, by the PBE method (Perdew–Burke–Ernzerhof) (Perdew and Wang, 1992; Perdew et al., 1996). The convergence of the Hellmann-Feynman forces is 0.01 eV/Å. The conjugate-gradient algorithm was employed for structural relaxations with an energy convergence criteria of 1 × 10^−5^ eV. The post-processing of figures were conducted by VESTA. In our calculations, Van der Waals (VDW) corrections are included to describe long-range interactions with the DFT-D2 method (Grimme, 2006). To avoid the interactions between the periodic slabs along the *z*-axis, the vacuum layer with the magnitude of 20 Å was inserted. Bader analysis was employed to estimate the amount of charge transferred among the pristine g-C_3_N_5_ surface and Li atoms (Bader, 1990). And, a 3 × 3 × 1 Gamma-centered k-point grid was used for sampling within the Brillouin zone (Monkhorst and Pack, 1976; Chadi, 1977).

The thermodynamics stability of Li@C_3_N_5_ was investigated by first-principles molecular dynamics (MD) simulations under the canonical (NVT) ensemble (Martyna et al., 1992). The adsorption energy of Li atom upon g-C_3_N_5_ were obtained by the equation below:

$$E_{ad}\left(\text{Li}\right)=[E\left({\text{Li}}_{k}◦{\text{C}}_{3}{\text{N}}_{5}\right)-E\left({\text{C}}_{3}{\text{N}}_{5}\right)-kE\left(\text{Li}\right)]/k,$$ (1)

where *k* represents the number of Li atoms, and *E* is the energy term. The averaged adsorption energy per hydrogen molecule was calculated by the following equation:

$$E_{ad}\left({\text{H}}_{2}\right)=[E\left(n{\text{H}}_{2}•{\text{Li}}_{k}◦{\text{C}}_{3}{\text{N}}_{5}\right)-E\left({\text{Li}}_{k}◦{\text{C}}_{3}{\text{N}}_{5}\right)-nE\left({\text{H}}_{2}\right)]/n,$$ (2)

where *n* indicates the number of adsorbed hydrogen molecules upon the surface of Li@C_3_N_5_. The hydrogen storage capacity can be obtained by the following equation:

$$\text{HSC}=\frac{\text{m}\left(\text{H}\right)*\text{M}\left(\text{H}\right)}{\text{m}\left(\text{C}\right)*\text{M}\left(\text{C}\right)+\text{m}\left(\text{N}\right)*\text{M}\left(\text{N}\right)+\text{m}\left(\text{Li}\right)*\text{M}\left(\text{Li}\right)}\times 100\text{\%},$$ (3)

where m(X) is the number of X (X = C, N, Li, and H) atom, and M(X) is the corresponding molar mass. The desorption temperature (T_D_) between hydrogen molecules and the substrate can be defined as:

$$T_{D}=\frac{E_{ad}}{K_{B}}{\left(\frac{\Delta S}{R}-lnP\right)}^{-1}.$$ (4)

Here, R and K_B_ are the universal gas constant and Boltzman constant, respectively; ΔS is the change of entropy for hydrogen molecules from the gas to liquid state (75.44 J mol^−1^ K^−1^); while P is the pressure of equilibrium (1 atm).

## 3 Results and discussion

### 3.1 Structural and electronic properties of Li-doped g-C_3_N_5_

**FIGURE 1:**
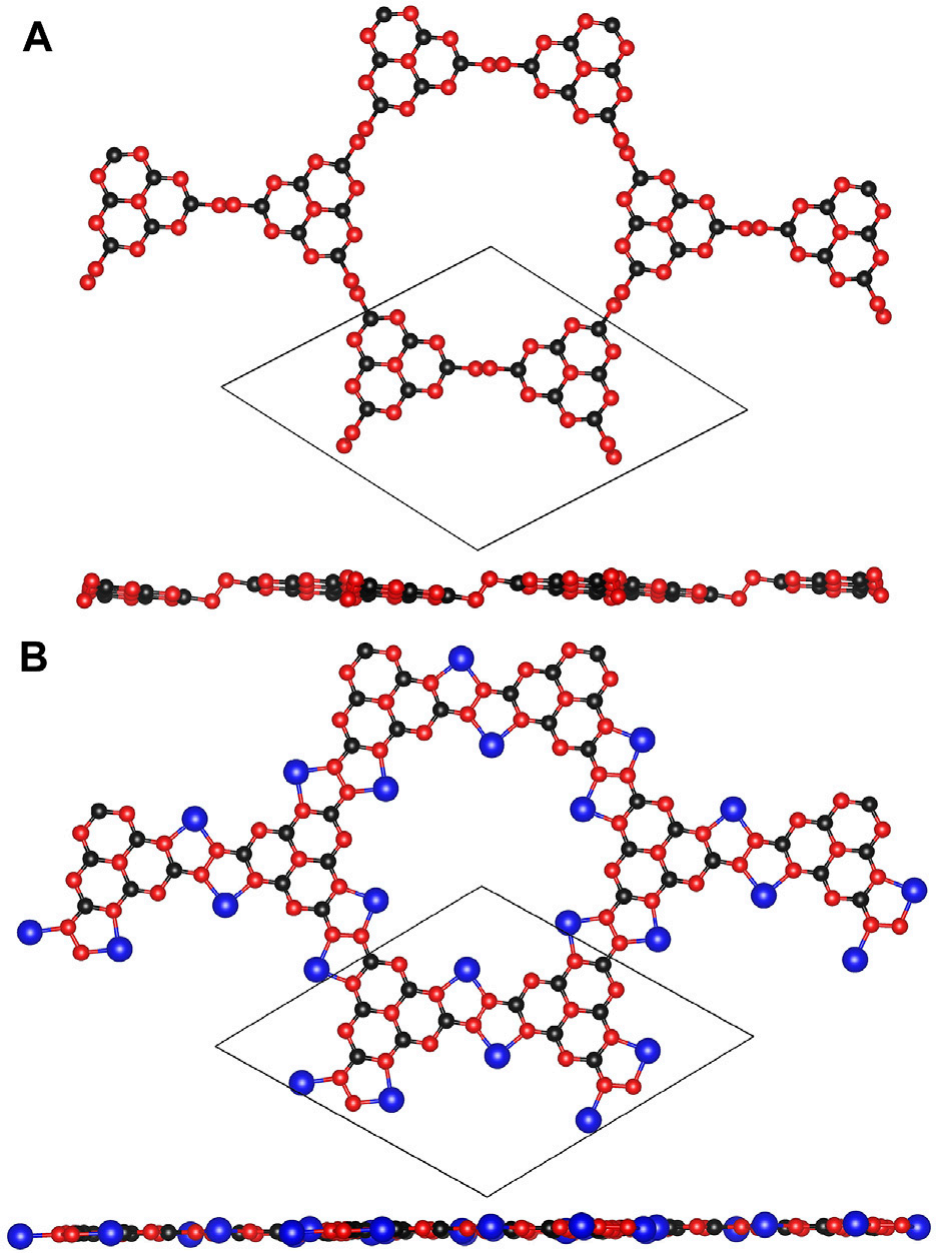
The optimized structures of **(A)** g-C_3_N_5_ primitive cell and **(B)** Li@C_3_N_5_ primitive cell. The black, red and blue balls represent C, N and Li atoms, respectively.

**FIGURE 2:**
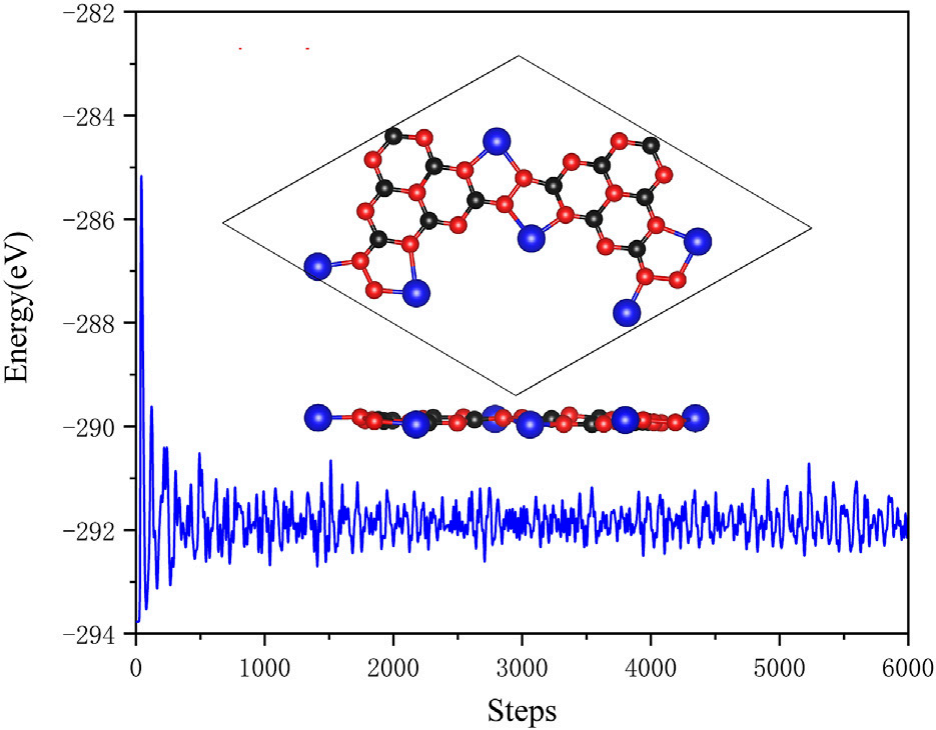
The AIMD simulations of Li@C_3_N_5_. The black, red and blue balls represent C, N and Li atoms, respectively.

The optimized configurations of pristine and Li@C_3_N_5_ were presented in Figure 1. To systematically estimate its thermodynamics stability, *ab initio* molecular dynamics (AIMD) simulations were conducted, and the results were shown in Figure 2 for reference. It is notable that under the temperature of 400 K, the fluctuation of the total energy mainly oscillates at the equilibrium point; and from the snapshot that reflects the complex structure of the last frame, we noticed that the **Li** atom can stably bind with the **N** atom of the g-C_3_N_5_. All the observations indicate the high stability of this complex 2D material. At the same time, the mechanical properties of the pristine g-C_3_N_5_ (see in Figure 1A) were also investigated, and the simulated independent elastic constants are: C_11_ = 17.05 N/m, C_12_ = 15.24 N/m, and C_66_ = 0.91 N/m, respectively. These obtained values satisfy the Born-Huang criterion, revealing that the such a **CN** bonding network displays considerable mechanical hardness.

**FIGURE 3:**
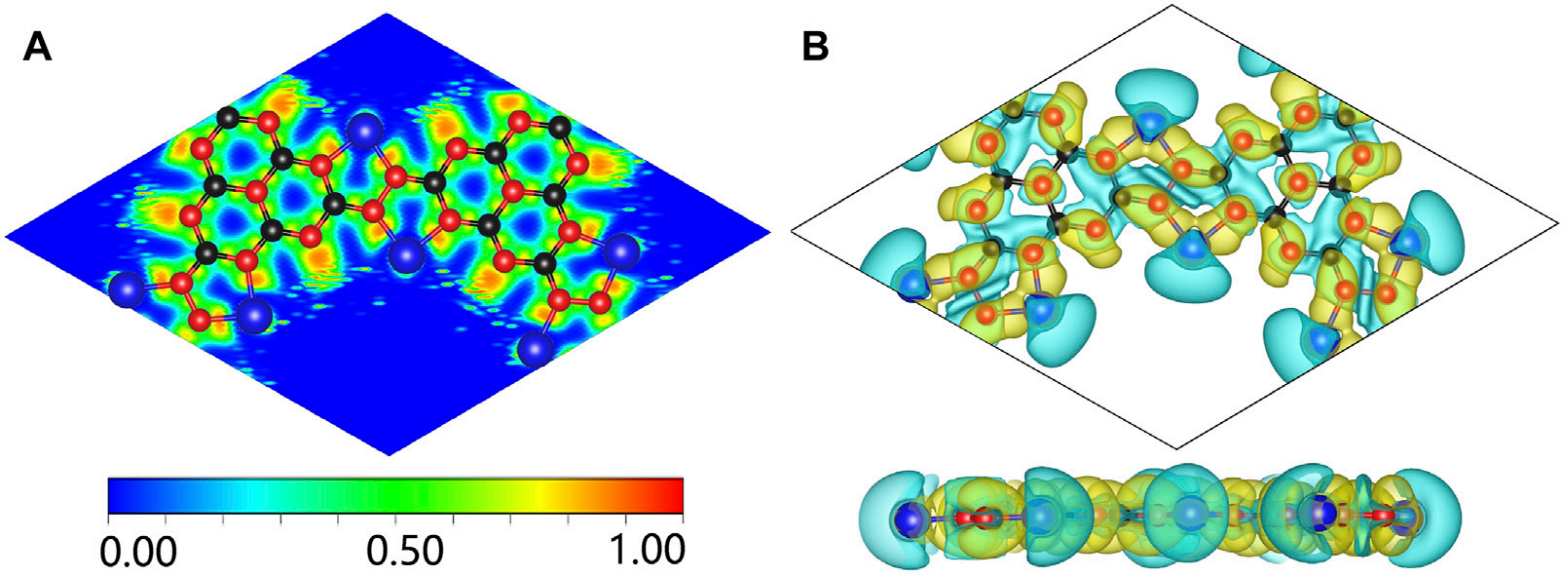
**(A)** The simulated ELF of Li@C_3_N_5_; the isosurface value is set to be 0.0013. **(B)** The calculated charge density difference of Li@C_3_N_5_. The black, red and blue balls represent C, N and Li atoms, respectively.

**FIGURE 4:**
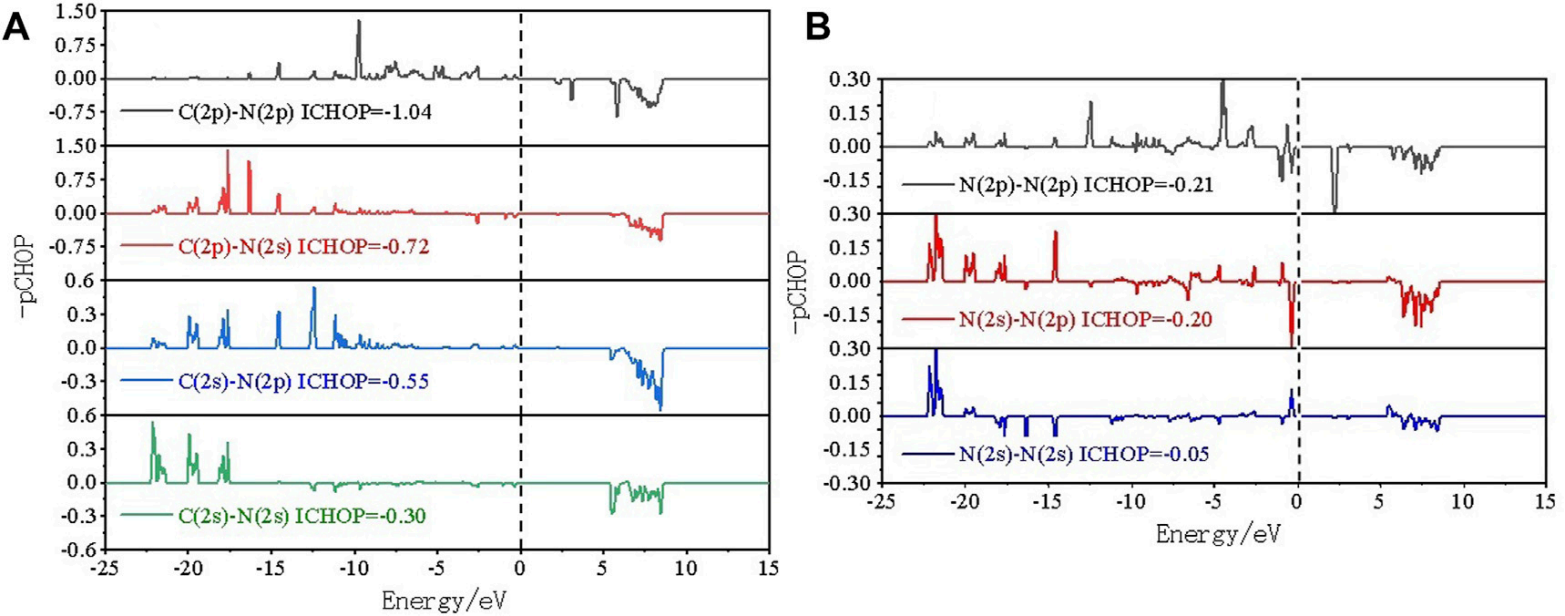
Crystal orbital Hamilton population COHP analysis of **(A)** C-N and **(B)** N-N bonds within pristine g-C_3_N_5_.

**FIGURE 5:**
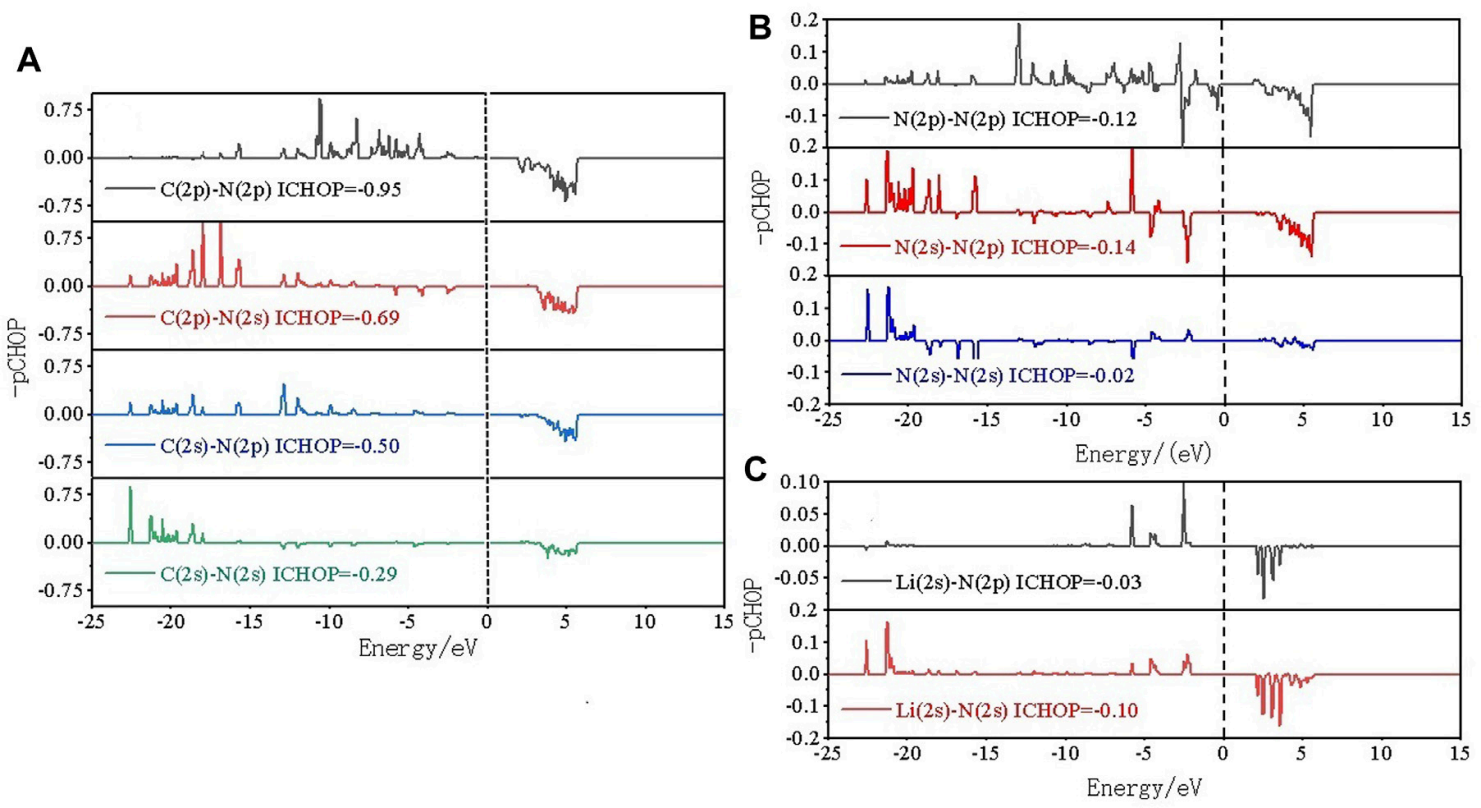
COHP analysis of **(A)** C-N, **(B)** N-N and **(C)** Li-N bonds within Li@C_3_N_5_.

**FIGURE 6:**
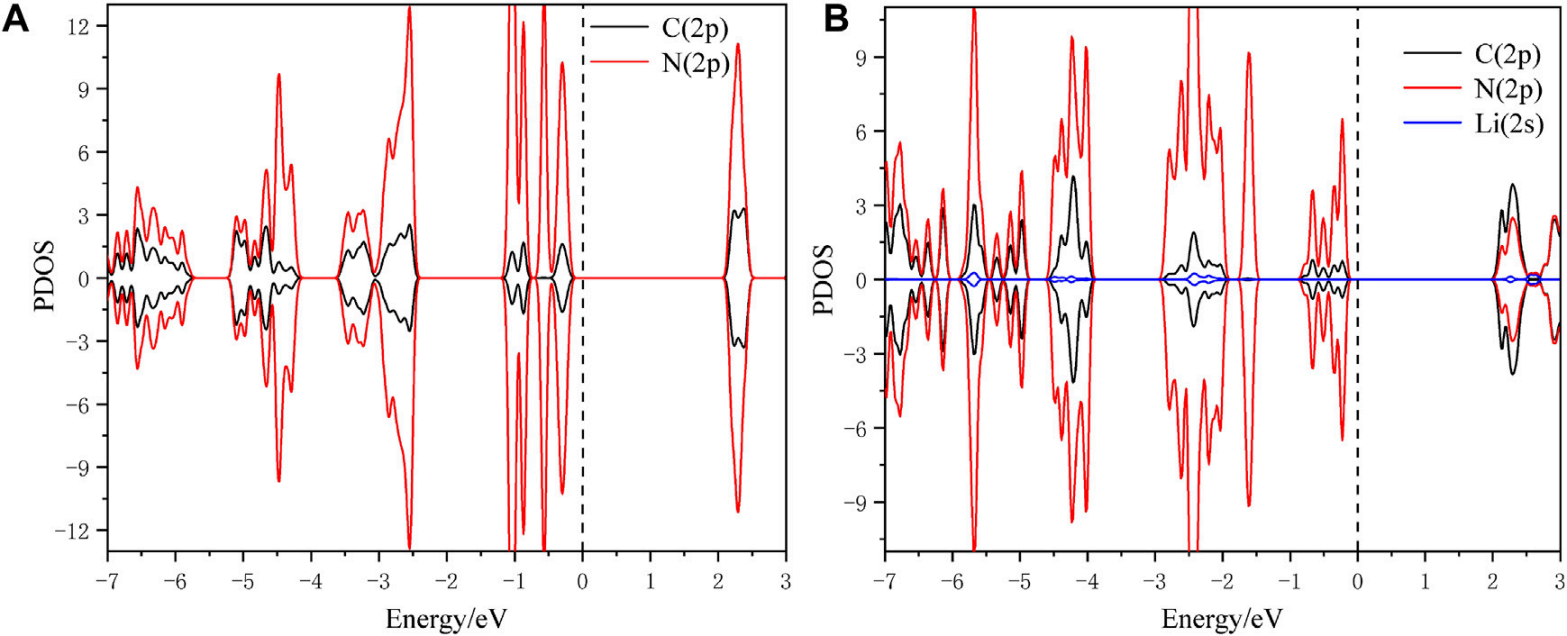
The simulated PDOS of **(A)** pristine g-C_3_N_5_ and **(B)** Li@C_3_N_5_.

To in-depth solve the binding mechanism of g-C_3_N_5_ monolayer, the electronic localization function (ELF) and charge density difference calculations were carried out, the results are presented in Figures 3A, B. The crystal face was obtained by cutting the g-C_3_N_5_ primitive cell based on the Mille index (−2.528, 1, 379.194). One can clearly see that the calculated ELF value of **N-C** binding is around 0.8 that is, within the reasonable range of covalent bonds. To further investigate the strength of this kind of binding, the Crystal Orbital Hamilton Population (COHP) calculations were employed, and the calculated results of **N-N** and **N-C** bonds are −0.46 and −2.61 eV, respectively (more details can be found in Figures 4A, B). With addition of Li atoms, we noticed that both the N-N (changed to −0.28 eV) and N-C (changed to −2.43 eV) bonds become weaker (more details can be found in Figures 5A–C), further indicating the fact that the charges of Li atoms are effectively transferred to the antibonding orbitals of C-N and N-N bonds. And for pristine g-C_3_N_5_, we notice the anitbonding states move down to the Fermi level, further indicating its capability of adsorbing metallic atoms that own ability of electron donating. The partial density of states (PDOSs) were also calculated for reference, and the results are shown in Figure 6. We can notice that the state density component of conduction band bottom and valence band top were both dominated by the **2p** orbitals of **N** atoms, while less contributions were from **2p** orbitals of **C** atoms.

The **N-2p** and **C-2p** orbitals overlap at the energy interval, indicating chemical interactions among these two atoms; such an observation is consistent with the conclusion obtained by ELF calculations. We hope these theoretical insights could be instructive for future experiment work upon this novel 2D material.

With the addition of Li atoms (the configuration is shown in Figure 1B), we first estimated the absorption energy per metal atom upon this 2D material; the obtained value is around −4.88 eV, within the reasonable range to overcome the risk of cohesive effects. Secondly, we notice that its **2s** orbital can effectively overlap with the **2p** orbitals of N atoms from g-C_3_N_5_ within the ranges of −2.5 ∼ −2 eV and −4.5 ∼ −4 eV, respectively. (details can be found from the simulated PDOSs shown in Figure 6), indicating the binding availability between these atoms. And also, from the calculated results of ELF, we can confirm that there exist chemical interactions between the added Li atoms and g-C_3_N_5_ monolayer. With the assistance of Barder analysis, we figure out each Li atom transfers 0.89 *e* ^−^ to g-C_3_N_5_, and displays electropositivty. Such an observation further indicates the possible potency of these metallic sites for gas molecules adsorption due to enhanced electrostatic interactions.

### 3.2 Hydrogen storage performance *in Li@*C_3_N_5_

**FIGURE 7:**
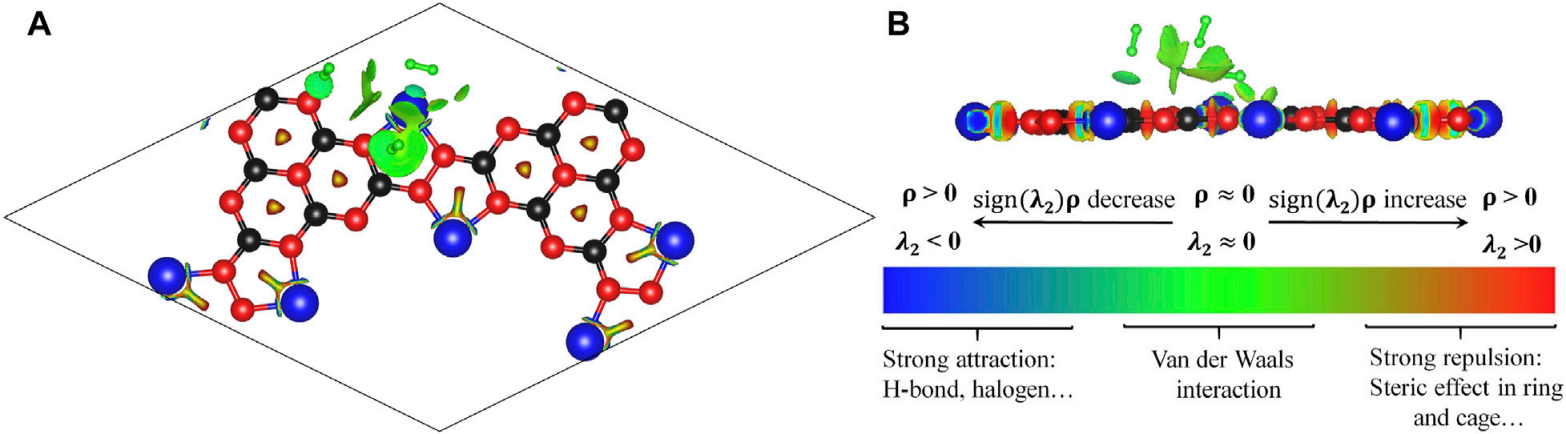
**(A,B)**. The simulated RDG of Li@C_3_N_5_ with adsorbed hydrogen molecules.

Then we optimized the geometric configurations of Li@C_3_N_5_ with adsorbed hydrogen molecules by first-principles calculations. We can clearly see that the adsorbed hydrogen molecules can be easily polarized by the metallic sites that display considerable electropositivity, consistent with our previous conclusion. We can also see from the calculated reduced density gradient (RDG) shown in Figure 7, the electrostatic interactions between these polarized hydrogen molecules and Li atoms can be correspondingly enhanced. And from the results shown in Figure 7, the main interaction between the adsorbed hydrogen molecules and Li atoms can be attributed to van der Waals interaction.

**FIGURE 8:**
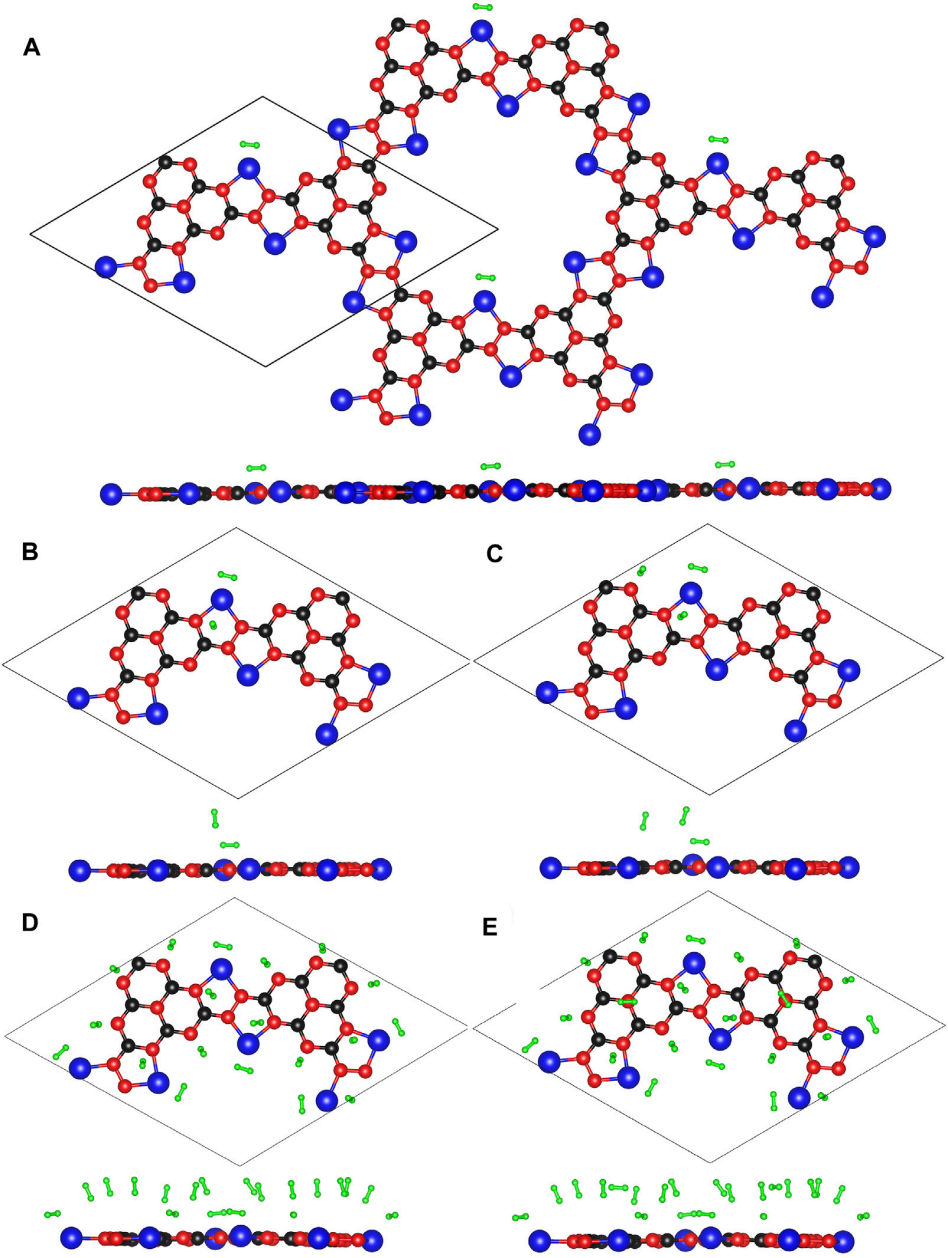
**(A–E)** The optimized configurations of Li@C_3_N_5_ with multiple adsorbed hydrogen molecules.

The optimized configurations of Li@C_3_N_5_ with multiple adsorbed hydrogen molecules are presented in Figure 8 for reference. The corresponding results are listed in Table 1. The averaged adsorption energy per H_2_ molecule is ranged from −0.22 to −0.13 eV, with the storage capacity from 0.43 to 8.65 wt%. And for the adsorbed hydrogen molecules, the binding length of **H-H** is predicted to be slightly stretched between 0.753 Å and 0.755 Å. As stated before, the behind reason can be attributed to the induced polarization by addition of metal atoms. We also anticipated that the storage capacity can be further increased, with the decoration of extra metal atoms. We conclude that the overall electronic structures of 2D monolayer materials can be favorably improved with respect to specific needs of adsorption tasks in real practice, further indicating a promising methodology by computational evaluation for functional materials design.

**TABLE 1:** The average adsorption energy E_*ad*_, the average bond length R_H-H_, the desorption temperature T_d_ and the hydrogen storage density (HSC) for Li@C_3_N_5_ monolayer.

Systems	E_ad_ (eV)	$R_{H-H}\left(\overset{{}^{\circ}}{A}\right)$	HSC (wt%)	T_d_ (K)
Li@C_3_N_5_-1H_2_	−0.22	0.755	0.43	283
Li@C_3_N_5_-2H_2_	−0.15	0.754	0.80	187
Li@C_3_N_5_-3H_2_	−0.13	0.754	1.29	167
Li@C_3_N_5_-18H_2_	−0.13	0.754	7.79	167
Li@C_3_N_5_-20H_2_	−0.13	0.753	8.65	167

## 4 Conclusion

In summary, we proposed a systematic methodology for 2D materials’ hydrogen storage performance evaluation; and we decently investigated the adsorption mechanism of Li@C_3_N_5_ monolayer. DFT computational studies reveal that the pristine g-C_3_N_5_ cannot form strong interactions with hydrogen molecules; by decoration of Li atoms, its structure and electronic properties can be modified. Binding interaction between the added Li atoms and the adjacent N atoms from g-C_3_N_5_ is proved to be stable. Bader charge analysis reveals that each Li atom can transfer 0.89 e^−^ to the pristine g-C_3_N_5_ and display considerable electropositivity. These metallic sites can essentially polarize the adsorbed hydrogen molecules and generate stronger electrostatic interactions, leading to the enhanced hydrogen adsorption performance. The average adsorption energy per hydrogen molecule on the Li@C_3_N_5_ complex 2D material is up to −0.22 eV that is, within a reasonable range; and each primitive cell can adsorb up to 20 hydrogen molecules, and the overall storage capacity can reach to 8.65 wt%. The insights obtained by this study indicated that the Li@C_3_N_5_ can serve as a promising storage media; and moreover, the proposed methodology is highly instructive for future studies.

## Data Availability

The raw data supporting the conclusion of this article will be made available by the authors, without undue reservation.
